# Water boatman survival and fecundity are related to ectoparasitism and salinity stress

**DOI:** 10.1371/journal.pone.0209828

**Published:** 2019-01-16

**Authors:** Vanessa Céspedes, Antonio G. Valdecasas, Andy J. Green, Marta I. Sánchez

**Affiliations:** 1 Departamento de Ecología de Humedales, Estación Biológica de Doñana, CSIC, Seville, Spain; 2 Departamento de Biodiversidad y Biología Evolutiva, Museo Nacional de Ciencias Naturales, CSIC, Madrid, Spain; 3 Instituto Universitario de Investigación Marina (INMAR), Campus de Excelencia Internacional/Global del Mar (CEI·MAR), Universidad de Cádiz, Puerto Real (Cádiz), Spain; Universidad de Sevilla, SPAIN

## Abstract

Salinity is increasing in aquatic ecosystems in the Mediterranean region due to global change, and this is likely to have an important impact on host-parasite interactions. Here we studied the relationships between infection by ectoparasitic water mites and salinity variation, on survival and fecundity of water boatmen Corixidae in the laboratory. Larvae of *Sigara lateralis* parasitised by larval mites (*Hydrachna skorikowi*) had lower survivorship, and failed to moult to the adult stage. In adult corixids (*S*. *lateralis* and *Corixa affinis*) fitness was reduced at high salinities and in individuals infected by *H*. *skorikowi*, both in terms of survival and fecundity. We also found evidence for parasitism-salinity interactions. Our results suggest that ongoing increases in salinity in Mediterranean ponds due to climate change and water abstraction for agriculture or urban use have a strong impact on water bugs, and that their interactions with ectoparasites may modify salinity effects.

## Introduction

The ecological consequences of being parasitized and the net fitness cost for the host critically depend on environmental conditions. Parasites often interact in complex ways with other stressors (reviewed in [1]), and the outcome of the interaction can be positive or negative for the host. The parasite and the stressor may often have additive or synergistic effects. For example, Kelly et al. [2] showed that the herbicide glyphosate and the trematode parasite *Telogaster opisthorchis* Macfarlane, 1945, act synergistically on survival and the development of spinal malformations in juvenile *Galaxias anomalus* Stokell, 1958. Less often, parasites can ameliorate the effect of the stressor. For example, Sánchez et al. [3] showed that cestode parasites increased brine shrimp resistance to metalloid arsenic, by enhancing the host antioxidant defences. The outcome of the interaction between different stressors also depends on the particular life-history trait considered. For example, Coors and De Meester [4] experimentally evaluated joint effects of parasites, predation and contaminants on *Daphnia magna* Strauss, 1820, and found a full range of combined effects–additive, synergistic and antagonistic–depending on the trait considered, and demonstrated that multiple stressors reduced population growth by up to 28%.

Parasitism should therefore be studied in the context of key environmental stressors. Particularly important is interaction with climate change, which is predicted to have important effects on parasitism, disease transmission and possibly virulence, with a major impact in aquatic ecosystems [5]. On the other hand, we need to integrate studies across multiple species and developmental stages. For example, infection data from adult stages may underestimate the impact of parasites in natural populations, because early developmental stages are generally more sensitive [6]. Many studies have focused on vector- and food-borne diseases affecting humans, livestock, or domestic animals [7–10]. However, less attention has been devoted to host-parasite interactions that do not involve humans [5].

One interesting model system for studying interactions between environmental stress and parasitism is water mites infecting aquatic insects. Water mites or Hydrachnidia are the most diversified group of the Acari in freshwater ecosystems, with more than 300 genera and 6000 species [11, 12]. They occur in almost all fresh and brackish aquatic environments around the world where they can reach densities exceeding 2000 specimens per square meter [13]. Water mites have a complex life cycle involving ecto-parasitic and free living stages [14]. While the deutonymph (second larval stage) and adult instars are typically free-living predators feeding mainly on insect eggs, insect larvae and microcrustaceans, the larval stage is morphologically distinct and ecto-parasitic [15]. Larvae mainly parasitize aquatic and semiaquatic insects, with relatively weak host-specificity [16]. They feed on the host haemolymph and after a period of engorgement they detach themselves. One of the most common genera is *Hydrachna*, which often parasitize water boatmen (Heteroptera: Corixidae). Corixids are key links in aquatic food webs being both primary and secondary consumers, and serving as food for predatory vertebrates such as fish and birds [17, 18]. Water mites can strongly impact their host populations and influence biological interactions between corixid species [19, 20].

Mediterranean temporary ponds, such as those of Doñana National Park (south-west Spain), allow the study of interactions between water mites and corixids in conjunction with environmental stressors. In Doñana, *Hydrachna skorikowi* Piersig, 1900, is one of the most abundant and widespread watermites [20]. Water salinity is a key natural stressor in arid and semiarid zones of the Mediterranean basin. It has a major influence on biotic communities and is affected by a range of natural and anthropogenic processes. During the summer, temperature and salinity increase, with the concomitant reduction of depth and surface area in Doñana ponds, which completely dry out by August [21]. In these conditions, corixid densities become high and allow for increasing contact between ectoparasites and their hosts, although salinity exerts strong controls on aquatic communities, eliminating sensitive species [22, 23]. Human water use and climate change in Doñana and rest of the Mediterranean region tend to increase salinities [24, 25].

In the present work, we used laboratory experiments to study the interactions between corixids and water mites and how they were affected by environmental stressors. We examined the effect of salinity on adult corixids (*Sigara lateralis* Leach, 1817 and *Corixa affinis* Leach, 1817) and how the results depend on the presence of ectoparasites (*H*. *skorikowi*). The salinity gradient is known to structure corixid communities in Mediterranean wetlands [26], but the role of parasitism has not previously been explored. We compared both adult corixid mortality and female fecundity (number of eggs and hatching success) between unparasitized individuals and those infected by mites. We also examined the relationship between the presence of *H*. *skorokowi* and the mortality rates of *S*. *lateralis* larvae.

## Methods

### Study area

Doñana is a Mediterranean wetland complex, situated on the Atlantic coast of south-west Spain (36°58'41''N, 6°20'40''W). It has the highest degree of environmental protection in Spain (National Park) and is one of the most emblematic protected areas in Europe. It was designated a UNESCO Man and Biosphere Reserve in 1980, a Ramsar Site (Wetland of international importance) in 1982, and a UNESCO World Heritage Site in 1994 [25]. The climate is sub-humid, with a well-defined seasonality. The area has very dry and hot summers and wet and cool winters (with rainfall mostly occurring between October and March). The region includes a rich network of more than 3000 temporary dune ponds that vary greatly in size, hydroperiod and salinity [25]. It also includes a few permanent and semipermanent lagoons formed in an area where discharges of adjacent dune and regional aquifers coincide [27]. The ionic composition of Doñana pond waters is dominated by Chloride (Cl^-^) and Sodium (Na^+^) as a result of the solubilisation of salts from the sediment and airborne marine salt deposition [21, 28]. Calcium, magnesium, sulphate and silica are usually present at much lower concentrations. The study was conducted on corixids and mites collected in one of the larger semi-permanent oligohaline lagoons of Doñana National Park, Laguna Dulce (see López et al. [29] for a limnological description).

### Study model

Twelve species of water boatmen Corixidae can be found in Laguna Dulce, which is representative of larger dune ponds in Doñana (see Florencio et al. [30], plus S1 and S2 Tables for more details), but only a fraction of them are regularly encountered. *Sigara lateralis* and *Corixa affinis* are the most common species of their respective genera (see S2 Table). Corixids used in our study are mainly omnivorous [31, 32]. Their piercing-sucking mouthparts allow them to feed on both plants and animals. They are known to be predators on other invertebrates such as cladocera or *Artemia* [32–34]. However, larvae generally feed at lower trophic levels, consuming much periphyton and phytoplankton [35–37]. Depending on the species and the availability of different foods, corixids can be more or less herbivorous (see Coccia et al. 2016 [37] for niche differences in different ponds).

Corixids from Doñana are commonly parasitized by two water mite species: *Hydrachna skorikowi* (Family Hydrachnidae) and *Eylais infundibulifera* (Koenike, 1897; Family Eylaidae) (see S3 Table and [20]). Larvae of Hydrachnidae are strictly aquatic and can use dissolved oxygen from the water; besides Nepomorpha (Heteroptera), they also parasitize other aquatic insects such as Dytiscidae and Hydrophilidae (Coleoptera) [38]. *Eylais* larvae are aerial [39], requiring an air-oxygen supply to survive, and are restricted to areas such as under the wings, tergites, the underside of the elytra and hemelytra of the host [40, 41].

### Experiments

Samples of corixids of different stages (larvae and adults, and both infected by *H*. *skorikowi* and uninfected) were collected with a hand net of 250 μm mesh in June 2014. Junta de Andalucía provided permission to work in Doñana National Park (Authorization number 2015107300003028). All specimens were transported to the laboratory in containers filled with water from the lagoon for subsequent experiments.

#### Experiment with corixid larvae

The objective of this experiment was to explore the relationship between water mite infection and larval mortality. We were unable to include a salinity gradient due to the shortage of infected larvae required for testing multiple experimental factors. On 9 June, we collected larvae of *S*. *lateralis* parasitized with a single *H*. *skorikowi* (mite identification was confirmed later, see below) and unparasitized individuals from Laguna Dulce. We collected a total of 30 instar II, 30 instar III, 35 instar IV and 35 instar V of unparasitized *S*. *lateralis*, and 13 instar II, 11 instar III, 15 instar IV and 21 instar V of parasitized *S*. *lateralis*. All the larvae were individually placed in plastic containers with aquarium stones and sterilized water from the collection area (350 ml). To minimize mortality of this particularly vulnerable stage, individuals were placed in a climatic chamber under conditions simulating the natural environment (25°C and 12:12 photoperiod). On alternate days, we added 1 ml of algae (*Tetraselmis chuii*–Easy Algae) as food. The water level of each container was checked and adjusted every day. Oxygen level was measured regularly, always being between 93–98% (saturation). During 15 days we daily checked for mortality of corixid individuals. From day 15 we checked for moulting to adult stage until the last individual moulted (30 days). We removed those individuals for which mites moulted and became detached from the host (5 in total).

#### Experiment with adult corixids

The objective of this experiment was to explore the relationship between mite infection and adult mortality and female fecundity (number of eggs and hatching success) under different salinity conditions. We selected adult *Sigara lateralis* and *C*. *affinis* parasitized with a single *Hydrachna skorikowi*, or unparasitized adults, from Laguna Dulce. We rejected individuals infected with more than a single mite so as to simplify the experimental design.

Corixids were acclimatized for 48 hours in a climate chamber at 20°C and 12h/12h photoperiod. These conditions were selected to make results comparable with other studies (for example Kefford et al. [6]). Each individual was placed in a 250ml container with a mesh (10 mm^2^ size and 1mm^2^ core) serving as substrate for eggs, and randomly allocated to one of 4–5 salinity treatments (depending on the corixid species). According to the natural conductivity range of the habitat commonly occupied by selected species (including lower and upper extreme values [2, 20]), salinity treatments were: 0.5, 5, 10 and 15 g/l for *S*. *lateralis* and 0.5, 5, 10, 15 and 20 g/l for *C*. *affinis*. We used 10 *S*. *lateralis* and 5 *C*. *affinis* for each combination of salinity treatment, sex and parasitic status (a total of 160 *S*. *lateralis* and 100 *C*. *affinis*). The different saline solutions were prepared by dissolving marine salt (Ocean Fish, Prodac, Citadella, Italy) in distilled water. We also used a control group with water from the lagoon where corixids were collected (0.8 g/l). It included 40 females and 40 males (20 parasitized and 20 unparasitized of each sex) of *S*. *lateralis* and the same for *C*. *affinis*.

Corixids were fed every day with 2 frozen chironomids each. The water level of each container was checked and adjusted every day. Mortality was checked every day, and the number of eggs produced by *S*. *lateralis* within two weeks counted with the aid of a stereomicroscope. *C*. *affinis* did not reproduce in the laboratory. Hatched larvae were also counted during a month, to estimate hatching success. Experiments were run for 15 days for *S*. *lateralis*, and 35 days for *C*. *affinis*. This difference is due to the greater longevity of the larger *C*. *affinis* in the laboratory. We removed those individuals (three *S*. *lateralis*) for which mites moulted and became detached from the host.

### Water mite identification

Prior to identification, water mite larvae were detached from the host under a Bausch and Lomb stereo microscope. Subsequently, larvae were mounted and studied with a Leica TCS SPE Laser Confocal Scanning Microscope (see Lorenzo-Carballa et al. [42] for detailed procedure). Optical serial sections were acquired and processed with Fiji/ImageJ (version 1.48d; downloaded from http://fiji.sc/Fiji), Amira (version 5.5.0) and Photoshop CS5 extended. The larvae of *Hydrachna skorikowi* are identifiable by the similar length of lateral border of coxa I and II, and the presence of a ‘thick cone’ of setae on coxa III [43].

### Statistical analysis

Cox regression models were used to examine the survival of corixids (time to death from the start of the experiment) in relation with water mite infection and larval stage. The interaction stage x infection status was also included in the model. We also used cox regression models to analyse the survival time of *Sigara lateralis* adults in relation to salinity (0.5, 5, 10 and 15 g/l together with water from the collection site as a control (0.8 g/l)), infection status (infected by *H*. *skorokowi* or uninfected), corixid sex and their interactions. A similar cox regression analysis was conducted for *C*. *affinis* adults, incorporating the additional salinity treatment of 20 g/l (S4 Table and S2 Fig).

GLMs were used to analyze the number and hatchability of *S*. *lateralis* eggs in relation with salinity (0.5, 5, 10 and 15 g/l, plus control water from the collection site (0.8 g/l)) and parasitic status (infected by *H*. *skorikowi* or uninfected) within two weeks. A Poisson error distribution with a log link function was applied for the number of eggs, and a binomial error distribution and logit link function for the percentage of hatching. Overdispersion were checked for and corrected with Pearson Chi-square.

All statistical analysis were performed using R Version 1.1.453 using survival and survminer packages, and Statistica 13.3 software.

## Results

### Experiments with corixid larvae. Association between mite infection and larval mortality

None of the parasitized larvae (n = 60) reached the adult stage, compared to 43.1% of unparasitized larvae (n = 130), a highly significant difference (Fisher exact, P < 0.001). On four occasions the corixid individual died at stage V during a moult as shown in S1 Fig, although the mites completed development to the adult stage. On 4 occasions, infected larvae at stages II to IV did moult successfully to the next instar but did not reach the adult stage.

**Fig 1:**
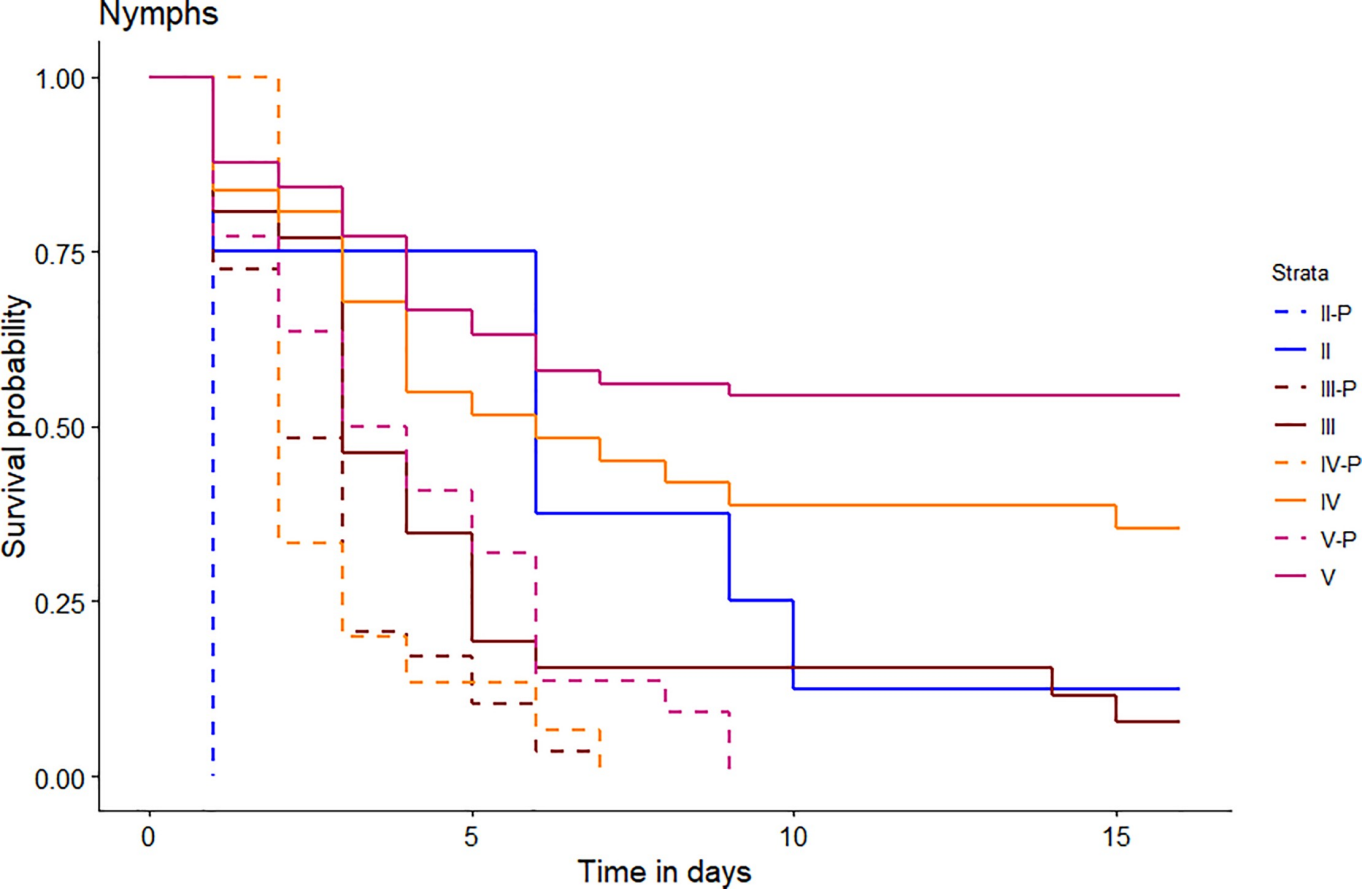
Comparison of cumulative survival of different stages (II, III, IV and V) of larvae of *Sigara lateralis* between those parasitized by *Hydrachna skorikowi* and those unparasitized. Individuals were maintained in water from the collection site (Dulce pond, 0.8 g/l). Stages represented are those for the beginning of the experiment, e.g. if a larva moulted from stage II to III before death, it is represented here as II.

Survival time in the laboratory was therefore shorter for parasitized larvae (mean ± SE, 2.957 ± 0.244, range 1–8 days) than for unparasitized larvae (8.544 ± 0.564, range 1–15 days; Fig 1).

Cox proportional hazard regression analysis showed a negative significant correlation between infection and larval survival time (Table 1). There was also a significant effect of larval stage (Table 1), uninfected larvae of instar V being more likely to survive than earlier instars (Table 1). The interaction parasitic status x larval stage was also statistically significant (Table 1).

**Table 1:** Results of Cox proportional hazard regression analysis on *S*. *lateralis* larval survival based on different parasitic status and developmental stages. The table shows for each term in the design matrix the estimated coefficient $\beta_{\hat{j}}$ (coef), the relative risk exp ($\beta_{\hat{j}}$) (exp (coef)), the standard error, the *z*-value and the corresponding *P*-value. Each *P*-value provides a test for the difference of each level with respect to the baseline. The overall *P*-value for factors with more than two levels (i.e. stage) and for the interaction stage*infection status, is obtained through the Wald test and is shown under the table.

Effect	Level of effect	coef	exp (coef)	s.e. (coef)	Z-value	P-value (>|z|)
Infection status	Unparasitized	-3.045	0.047	0.678	-4.48	**P<0.001**
Stage	III	-1.627	0.196	0.558	-2.91	**0.0035**
	IV	-1.673	0.187	0.585	-2.85	**0.0042**
	V	-2.123	0.119	0.574	-3.69	**P<0.001**
Stage*infection status	III*unparasitized	2.538	12.651	0.725	3.49	**P<0.001**
	IV*unparasitized	1.645	5.184	0.746	2.20	**0.027**
	V*unparasitized	1.430	4.180	0.731	1.96	**0.047**
	Concordance = 0.717 (s.e. = 0.03). Rsquare = 0.379 (max possible = 1)					

Overall P-value for Stage variable and Stage* infection status interaction. Wald test “Stage”; X^2^ = 14.1, df = 3, P (> X^2^) = **0.0028** and “Stage* infection status”; X^2^ = 16, df = 3, P (> X^2^) = **0.0011**.

### Experiments with adult corixids. Mortality rate and fecundity in relation with mite infection and salinity

**Fig 2:**
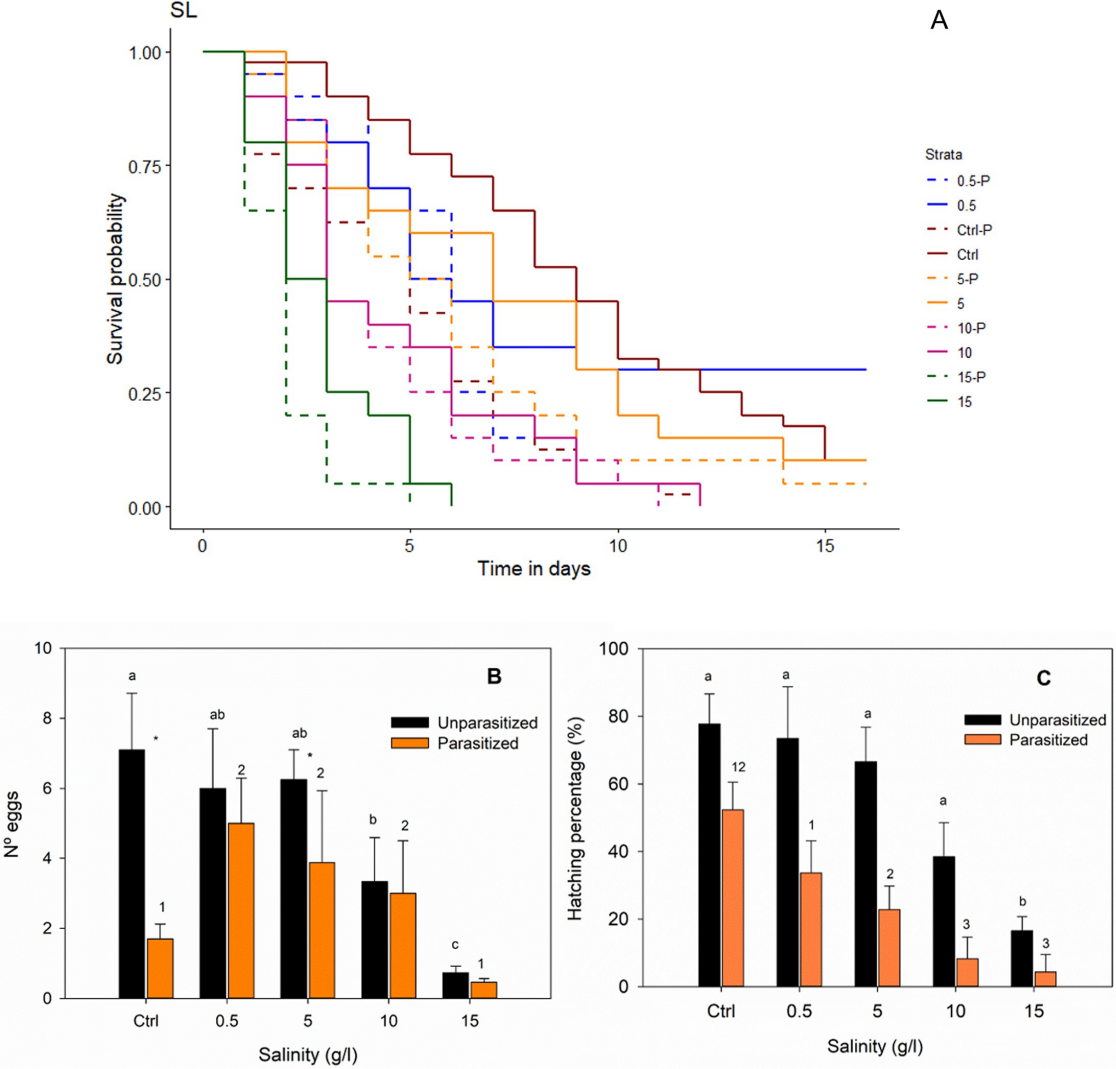
(A) Survival time (cox regresion model), (B) number of eggs (GLM with a Posisson error and log link function) and (C) hatching percentage (GLM with a Binomial error and logit link function) for adult *Sigara lateralis* with and without mite parasites (*Hydrachna skorikowi*) under different salinity treatments. C.W. represents water from the collection site (0.8 g/l). Different letters above bars indicate significant differences (p ≤ 0.05 after Bonferroni correction) for unparasitized groups, numbers above bars indicate significant differences for parasitized groups and “*” between bars indicate significant differences for the interaction Infection status*salinity treatment.

Cox regression analysis showed both mite infection by *H*. *skorikowi* and high salinities to be negatively associated with survival time of adult *S*. *lateralis* (Fig 2A, Table 2). Survival time was significantly higher for females than males. There were also a significant interaction between salinity and infection status, indicating a relatively higher mortality of infected boatmen at higher salinities. Interations between infection status and sex and between salinity and sex were also significant, indicating a greater mortality in infected females, and in males exposed to high salinity, respectively (Table 2).

**Table 2:** Effects of salinity treatments, infection status by *Hydrachna skorikowi*, sex (female and male) and their interactions on survival times in *Sigara lateralis* adults (Cox regression analysis). Salinity treatments were 0.5, 5, 10 and 15 g/l, and water from the collection site as a control (Ctrl: 0.8 g/l). The table shows for each term in the design matrix the estimated coefficient $\beta_{\hat{j}}$ (coef), the relative risk exp ($\beta_{\hat{j}}$) (exp (coef)), the standard error, the *z*-value and the corresponding *P*-value. Each *P*-value provides a test for the difference of each level with respect to the baseline. The overall *P*-value for factors with more than two levels (i.e. salinity) and interactions infection status*sex, sex*salinity are obtained through the Wald test and are shown under the table.

Level	Level of effect	coef	exp (coef)	s.e. (coef)	Z-value	P-value (>|z|)
Infection status	Unparasitized	-0.889	0.411	0.373	-2.388	**0.016**
Sex	male	1.459	4.301	0.371	3.917	**P<0.001**
Salinity	Ctrl	0.955	2.598	0.374	2.551	**0.010**
	5	-0.218	0.804	0.417	-0.522	ns
	10	0.565	1.759	0.418	1.350	ns
	15	2.738	15.452	0.429	6.369	**P<0.001**
Infection status*sex	Unparasitized * male	0.461	1.586	0.267	1.721	**P<0.001**
Infection status*salinity	Unparasitized * Ctrl	-0.719	0.538	0.403	-2.535	**0.010**
	Unparasitized * 5	-0.619	1.388	0.465	0.705	ns
	Unparasitized * 10	0.782	1.957	0.466	1.994	**0.049**
	Unparasitized * 15	0.672	0.826	0.489	-0.390	ns
Sex*Salinity	Male* Ctrl	-1.638	0.194	0.423	-3.872	**P<0.001**
	Male* 5	0.4003	1.492	0.461	0.870	ns
	Male* 10	0.2400	1.271	0.473	0.508	ns
	Male* 15	-1.121	0.326	0.493	-2.277	**0.02**
	Concordance = 0.78 (s.e. = 0.026). Rsquare = 0.525(max possible = 1)					

Overall P-value for Salinity variable and Infection status*Salinity and Sex*salinity interactions. Wald test “Salinity”; X^2^ = 26.3, df = 4, P (> X^2^) **<0.0001**; “Infection status*Salinity”; X^2^ = 14.8, df = 4, P (> X^2^) = 0.0051; “Sex*Salinity”; X^2^ = 35.5, df = 4, P (> X^2^)) **<0.0001**.

The Cox regression analysis for *C*. *affinis* showed significantly higher survival times for uninfected individuals and salinities below 10 g/l (S4 Table, S2 Fig). There was also a significant interaction between infection status and sex, indicating a greater mortality in infected females. The interaction infection status x salinity was marginally significant, reflecting a trend towards greater mortality in parasitised corixids at higher salinities (S4 Table).

GLM analysis showed a significant relationship between egg production and parasitism, salinity and the interaction between the two factors (Table 3). The number of eggs produced by female *S*. *lateralis* within two weeks was lower in infected individuals and was reduced by high salinities ≥ 10 g/l (Fig 2B). The interaction reflects how the difference between unparasitized and parasitized individuals was strongest in the collection pond water, and almost disappeared at high salinities ≥ 10 g/l (Fig 2B).

**Table 3:** Results from GLMs of the effects of salinity and parasitic status on the number of eggs laid by *Sigara lateralis* within a week, using a Poisson error distribution and a log link function. Salinity treatments were of 0.5. 5. 10 and 15 g/l) plus control water from the collection site (0.8 g/l). Parasitized individuals were infected by a single *Hydrachna skorikowi*.

Effect	Level de Effect	Estimate	SE	df	Wald Stat	Pr (>|w|)
Intercept		0.977	0.102	1	92.003	**P<0.001**
Infection status	Unparasitized	0.211	0.102	1	4.284	**0.038**
Salinity	Ctrl	0.268	0.164	4	2.66	**P<0.001**
	5	0.616	0.195		9.967	** **
	10	0.174	0.198		0.775	** **
	15	-1.53	0.235		41.43	** **
Infection status*Salinity	Unparasitized*Ctrl	0.504	0.195	4	0.025	**0.023**
	Unparasitized*5	0.028	0.197		0.638	
	Unparasitized*10	-0.158	0.235		0.004	
	Unparasitized*15	0.015	0.165		9.377	

GLM analysis showed that egg hatchability was significantly lower in infected individuals. The effect of salinity and the interaction between salinity and infection status were not significant (Table 4, Fig 2C).

**Table 4:** Results from GLMs of the effects of salinity and parasitic status on hatchability of eggs laid by *Sigara lateralis* within a week, using a Binomial error distribution and logit link (0 = did not hatch; 1 = hatched). Salinity treatments were of 0.5. 5. 10 and 15 g/l) plus control water from the collection site (0.8 g/l). Parasitized individuals were infected by a single *Hydrachna skorikowi*. Estimates for “parasitized” and “0.5 g/l” are not included because they were aliased, but they are effectively zero.

Effect	Level de Effect	Estimate	SE	df	Wald Stat	Pr (>|w|)
Intercept		-0.453	0.426	1	1.131	0.287
Infection status	Unparasitized	-1.644	0.533	1	9.511	**0.002**
Salinity	Ctrl	-2.043	0.797	4	19.23	**P<0.001**
	5	-0.747	0.674			
	10	1.623	0.897			
	15	3.54	0.847			

## Discussion

We found that water boatmen infected with ectoparasitic larvae of aquatic mites consistently had lower survival and fecundity, and that infected larvae failed to reach the adult stage. We found that the salinity gradient also influences the survival of adult boatmen, with significant interactions between the presence of parasites and salinity. However, as in many studies of parasite effects [3], we were forced to compare infected and uninfected individuals within the same population. We were unable to conduct experimental infections, partly due to the high mortality corixids typically experience in laboratory conditions. Therefore, we did not demonstrate a causal relationship between infection and mortality or low fecundity, only a correlation. We cannot rule out the possibility that weaker individuals from the population are more susceptible to infection and therefore have lower fecundity and higher mortality rate. However, based on the strength of the effects we recorded, and previous literature demonstrating negative effects of mite infection on hemiptera (for example see [44]), we suggest that the most important explanation for our results is likely to be a causal relationship between mite infection and reduced survival and fecundity of corixids. Our results lend support to the view that parasitism and environmental stress do not act in isolation but instead interact to determine the traits of free-living organisms [45].

### The possible impact of ectoparasitic water mites on corixid hosts

Most studies exploring the effect of water mite parasites on insects have been conducted in adult hosts ([44]; but see Lanciani and May [46] for the effect of parasites on larval growth). To our knowledge, we found the first evidence that water mite infection is associated with a prevention of corixid larvae from attaining adulthood. Some infected larvae were able to complete moults during their development, but not one was able to reach the adult stage, compared to over a third of uninfected larvae. This striking difference raises the question as to whether larvae are low quality hosts for larval mites, although the prevalence of water mites in *S*. *lateralis* larvae and adults was similar in the field (S3 Table). Being larger and able to fly, adults may provide more resources than larvae, and can also act as a vector for mite dispersal. On the other hand, they may have stronger immune defences [47].

In line with previous studies on other corixid species [44, 48], we found evidence for an important correlation between water mite infection and adult host survival and fecundity, suggesting a negative impact of mites on corixid populations. High intensity of infection has previously been reported to induce mortality [49], but our results show that a single larva is associated with mortality. Female *S*. *lateralis* infected with water mites also had much lower egg production and hatching success. Similar results were obtained by Davids and Schoots [48] who found infection by *Hydrachna conjecta* to reduce the number of eggs laid by *Sigara striata*, and to cause the total castration of *Cymatia coleoptrata*. These findings suggest an association between water mites and ovarian development [50].

The long duration of the *H*. *skorikowi* engorgement period may explain their negative impact on hosts. Deutonymphs and adults of *H*. *skorikowi* have also been shown to feed on corixid eggs [51], which may increase the impact on the host at the population level. Given the different susceptibility of different corixid species to water mite infection [20], our results suggest water mites can have an influence on interactions among corixid species, and hence the structure of aquatic insect communities. For example, mites may limit the invasion success of the American *Trichocorixa verticalis*, which is now widespread in our study area [20, 26, 52].

### Effect of salinity on corixid fitness, and possible interactions with parasitism

Environmental stressors interact with parasites in aquatic ecosystems, but this remains little studied. Most research has been carried out with vertebrates, especially fishes [53–55] and has focused on interaction between parasites and pollution (environmental parasitology, [1, 4, 56]. Data on simultaneous effects of parasites and salinity from aquatic invertebrates are very scarce (but see crustacean studies [57, 58]).

There have been previous studies of salinity tolerance of corixids in the absence of mites, including species used in this study. For example, it has been shown that *T*. *verticalis* is a euryhaline species with a well-developed ability of osmoregulation [59–61]. However *Sigara* species, such as *S*. *scripta*, are thought to be osmoconformers [62], like other corixid species that occur in hyposaline waters [63]. These species have hyperosmotic regulation in freshwater conditions up to a particular osmotic concentration of the external medium (the osmotic concentration of their haemolymph). Above that osmotic concentration, they become conformers, equalling the osmotic concentration of the haemolymph with that of the external water until a lethal concentration is reached [64].

To our knowledge, there is no previous information on the interaction between salinity and mite parasitism in aquatic insects. These two factors are both important in the structuring of aquatic insect communities. Smith [19] showed that the spatial distribution of two sympatric water boatmen was determined by the presence of water mites, which excluded one of them at low salinity. Sánchez et al. [20] found that the prevalence of *H*. *skorikowi* and *E*. *infundibulifera* in Doñana were negatively correlated with salinity, and argued that this relationship could partially explain the low abundance of the more parasite-sensitive exotic species *Trichocorixa verticalis* in low salinity habitats, to the benefit of native corixids.

In the present study, we found evidence that both mite infection and salinity stress are negatively related with host survival and fecundity. In the absence of the parasite, and consistent with other studies [61, 65], host fecundity and survival was higher at lower salinities (< 10 g/l). This is probably because osmoregulation has an energetic cost that increases with external osmolarity [66]. Carbonell et al. [61] found longer hatching times of *Sigara selecta* eggs at the upper limit of their salinity tolerance. Survival and fecundity of corixids parasitized by mites were both reduced even further at higher salinities. Apart from other physiological effects, damage inflicted by the parasite to the host integument when attaching and feeding on the haemolymph [67, 68] may make the insect more vulnerable to salinity stress. The statistical interactions between salinity and parasitism effects were significant for survival and number of eggs laid, although not for hatchability. However, in the case of eggs laid this interaction did not show a clear, consistent trend with salinity, since fecundity was particularly low for the water from the collection site, despite its low salinity (Fig 2). This is perhaps related to some unmeasured parameter of water quality.

## Conclusions

Ectoparasitism and salinity were negatively associated (both independently and in interaction) with corixid survival, fecundity and larval development. The impact of water mite parasites in freshwater communities may be more important than previously reported, taking into account the lethality of larval infections. Further efforts should continue to develop protocols to enable experimental infections in the laboratory to confirm that mites cause the observed low survival and fecundity of infected corixids. These protocols could then be used to investigate further questions, e.g. to compare the success and fitness of mites infecting larvae and adult hosts. Salinity is itself increasing through global change, particularly in Mediterranean wetlands [24, 25], and our results suggest this will influence future distributions and abundance of Corixidae and their ectoparasites. In particular, increasing salinities will favour halotolerant species such as the alien *T*. *verticalis* [26, 52]. However, given the context-dependent nature of environmental stress and the particular characteristics of host-parasite interactions, predicting these changes is difficult, and more experimental work is required (e.g. with mesocosms combining mite and host communities, and incorporating temperature modifications).

## Supporting information

S1 Table Physico-chemical field-data. Physico-chemical characteristics of Laguna Dulce from RBD (Doñana Biological Reserve) on 09/06/2014.
(DOCX)

S2 Table List of Hemiptera species sampled and their abundance. List of species of Hemiptera and the abundance of each species sampled from Dulce pond (RBD) on 09/06/2014.
(DOCX)

S3 Table Prevalence of water mites in adults and larvae of sampled corixids. Prevalence of water mites in adults and larvae of corixids that were sampled in the field on the same date and in the same locality as samples collected for experimental analyses (Dulce pond on 09/06/2014). H (*Hydrachna skorikowi*), E (*Eylais infundibulifera*)).
(DOCX)

S4 TableSurvival analysis (Cox proportional hazard regression) for *Corixa affinis* adults.Effects of salinity treatments, infection status (*Hydrachna skorikowi* and uninfected), sex (female and male) and their interactions on survival times in *Corixa affinis* adults. Salinity treatments were 0.5, 5, 10 and 15 g/l and water from the collection site as a control (0.8 g/l). The table shows for each term in the design matrix the estimated coefficient $\beta_{\hat{j}}$ (coef), the relative risk exp ($\beta_{\hat{j}}$) (exp (coef)), the standard error, the *z*-value and the corresponding *P*-value. Each *P*-value provides a test for the difference of each level with respect to the baseline. The overall *P*-value for factors with more than two levels (i.e. salinity) and for the interaction infection status*salinity, is obtained through the Wald test and is shown under the table.(DOCX)

S1 Fig A failed moult of a *Sigara lateralis* larva infected by a larval water mite. A failed moult of *Sigara lateralis* apparently due to the consequences of mite parasitism. The individual died in the act of moulting from larva stage V to the adult stage. The exuvia from a water mite *Hydrachna skorikowi* is highlighted in an orange box (this was reddish. but is discoloured after preservation in alcohol). The mite moulted successfully into a free-living adult. Credit: Vanessa Céspedes.
(TIF)

S2 Fig Survival time for adult female and male *Corixa affinis* (CA) with and without mite parasites. Survival time for adult female and male *Corixa affinis* (CA) with and without mite parasites *Hydrachna skorikowi* under different salinity treatments. C.W. represents water from the collection site (0.8 g/l).
(TIFF)

## References

[1] Sures B. Environmental parasitology Interactions between parasites and pollutants in the aquatic environment. 2008; 434–438 10.1051/parasite/2008153434 18814718

[2] Kelly DW, Poulin R, Tompkins DM, Townsend CR. Synergistic effects of glyphosate formulation and parasite infection on fish malformations and survival. Journal of Applied Ecology. 2010; 47(2) 498–504

[3] Sánchez MI, Pons I, Martínez-Haro M, Taggart MA, Lenormand T, Green AJ. When parasites are good for health: Cestode parasitism increases resistance to arsenic in brine shrimps. PLoS Pathogens. 2016; 12(3) e1005459 10.1371/journal.ppat.1005459 26938743 PMC4777290

[4] Coors A, De Meester L. Synergistic, antagonistic and additive effects of multiple stressors: predation threat, parasitism and pesticide exposure in *Daphnia magna*. Journal of Applied Ecology. 2008; 45(6) 1820–1828

[5] Marcogliese DJ. Implications of climate change for parasitism of animals in the aquatic environment. Canadian Journal of Zoology. 2001; 79(8) 1331–1352

[6] Kefford BJ, Dalton A, Palmer CG, Nugegoda D. The salinity tolerance of eggs and hatchlings of selected aquatic macroinvertebrates in south-east Australia and South Africa. Hydrobiologia. 2004; 517(1–3) 179–192

[7] Harvell CD, Kim K, Burkholder JM, Colwell RR, Epstein PR, Grimes DJ, et al Emerging marine diseases—climate links and anthropogenic factors. Science. 1999; 285(5433) 1505–1510 10498537 10.1126/science.285.5433.1505

[8] Rose JB, Epstein PR, Lipp EK, Sherman BH, Bernard SM, Patz JA. Climate variability and change in the United States: potential impacts on water-and foodborne diseases caused by microbiologic agents. Environmental health perspectives. 2001; 109 (Suppl 2) 211 11359688 10.1289/ehp.01109s2211 PMC1240668

[9] Marcogliese DJ. The impact of climate change on the parasites and infectious diseases of aquatic animals. Rev Sci Tech. 2008; 27(2) 467–484 18819673

[10] Sternberg ED, Thomas MB. Local adaptation to temperature and the implications for vector-borne diseases. Trends Parasitology. 2014; 30(3) 115–122 10.1016/j.pt.2013.12.010 24513566

[11] Gledhill T. Watermites predators and parasites. Freshwater Biology. 1985; 53 45–59

[12] Zhang ZQ, Fan QH, Pesic V, Smit H, Bochkov AV, Khaustov AA, et al Order trombidiformes reuter, 1909 Animal biodiversity: an outline of higher-level classification and survey of taxonomic richness. Zootaxa. 2011; 3148 129–138 10.11646/zootaxa.3703.1.1 26146682

[13] Smith IM, Cook DR, Smith BP. Water mites (Hydrachnidiae) and other arachnids In Ecology and Classification of North American Freshwater Invertebrates (Third Edition). 2009; (pp 485–586)

[14] Proctor HC, Smith IM, Cook DR Smith BP. Chapter 25—Subphylum Chelicerata, Class Arachnida, In Thorp and Covich's Freshwater Invertebrates (Fourth Edition), edited by James H Thorp and D Christopher Rogers, Academic Press, Boston 2015; pp599–660

[15] Böttger K. Types of parasitism by larvae of water mites (Acari: Hydrachnellae). Freshwater Biology. 1976; 6(6), 497–500.

[16] Di Sabatino A, Gerecke R, Martin P. The biology and ecology of lotic water mites (Hydrachnidia). Freshwater biology. 2000; 44(1), 47–62.

[17] Applegate RL, Kieckhefer RW. Ecology of Corixidae (Water Boatman) in Lake Poinsett, South Dakota. American Midland Naturalist. 1977; 1:198–208.

[18] Kortegaard L. An ecological outline of a moulting area of Teal, Vejlerne, Denmark. Wildfowl. 1974; 25(25) 134–142

[19] Smith BP. Water mite parasitism of water boatmen (Hemiptera: Corixidae). 1977; (Thesis doctoral, University of British Columbia)

[20] Sánchez MI, Coccia C, Valdecasas AG, Boyero L, Green AJ. Parasitism by water mites in native and exotic Corixidae: Are mites limiting the invasion of the water boatman *Trichocorixa verticalis* (Fieber, 1851)?. Journal Insect of Physiology. 2015; 19(3) 433–447

[21] Serrano Martín L, Reina Vázquez MM, Martín Farfán G, Reyes Bárbara I, Arechederra A, León D, et al The aquatic systems of Doñana (SW Spain): watersheds and frontiers. Limnetica. 2006; 25(1–2) 11–32

[22] Frisch D, Moreno-Ostos E, Green AJ. Species richness and distribution of copepods and cladocerans and their relation to hydroperiod and other environmental variables in Doñana, south-west Spain. Hydrobiologia. 2006; 556(1) 327–340

[23] Muhling BA, Gaitán CF, Stock CA, Saba VS, Tommasi D, Dixon KW. Potential salinity and temperature futures for the Chesapeake Bay using a statistical downscaling spatial disaggregation framework. Estuaries and Coasts. 2018; 41(2) 349–372

[24] Jeppesen E, Brucet S, Naselli-Flores L, Papastergiadou E, Stefanidis K, Noges T, et al Ecological impacts of global warming and water abstraction on lakes and reservoirs due to changes in water level and related changes in salinity. Hydrobiologia. 2015; 750(1) 201–227

[25] Green AJ, Alcorlo P, Peeters ET, Morris EP, Espinar JL, Bravo‐Utrera MA, et al Creating a safe operating space for wetlands in a changing climate. Frontiers in Ecology and the Environment. 2017; 15(2) 99–107

[26] Carbonell JA, Velasco J, Millan A, Green AJ, Coccia C, Guareschi S, et al Biological invasion modifies the co-occurrence patterns of insects along a stress gradient. Functional Ecology. 2017; 31: 1957–1968

[27] Díaz-Paniagua C, Aragonés D. Permanent and temporary ponds in Doñana National Park (SW Spain) are threatened by desiccation. Limnetica. 2015; 34(2) 407–424

[28] Serrano L, Toja J. Limnological description of four temporary ponds in the Doñana National Park (SW, Spain). Archiv für Hydrobiologie. 1995; 133(4), 497–516.

[29] López T, Toja JU, Gabellone NA. Limnological comparison of two peridunar ponds in the Donana National Park (Spain). Archiv fur Hydrobiologie Stuttgart. 1991; 120(3) 357–378

[30] Florencio M, Serrano L, Gómez-Rodríguez C, Millán A, Díaz-Paniagua C. Inter-and intra-annual variations of macroinvertebrate assemblages are related to the hydroperiod in Mediterranean temporary ponds. Hydrobiologia. 2009; 634(1) 167–183

[31] Murillo J, Recasens L. Hábitos alimentarios de *Sigara lateralis* (Heteroptera, Corixidae). Misc Zool 1986; 10:135–140

[32] Simonis JL. Predator ontogeny determines trophic cascade strength in freshwater rock pools. Ecosphere. 2013; 4:1–25

[33] Wurtsbaugh WA. Food-web modification by an invertebratepredator in the Great Salt Lake (USA). Oecologia. 1992; 89:168–175 10.1007/BF00317215 28312870

[34] Céspedes V, Sánchez MI, Green AJ. Predator–prey interactions between native brine shrimp *Artemia parthenogenetica* and the alien boatman *Trichocorixa verticalis*: influence of salinity, predator sex, and size, abundance and parasitic status of prey. PeerJ. 2017; 5, e3554 10.7717/peerj.3554 28713654 PMC5508811

[35] Campbell BC. The spatial and seasonal abundance of *Trichocorixa verticalis* (Hemiptera:Corixidae) in salt marsh intertidal pools. Can Entomol. 1979; 111:1005–1011

[36] Kelts LJ. Ecology of a tidal marsh corixid, *Trichocorixa verticalis* (Insecta, Hemiptera). Hydrobiologia. 1979; 64:37–57

[37] Coccia C, Fry B, Ramírez F, Boyero L, Bunn SE, Diz-Salgado C, et al Niche partitioning between invasive and native corixids (Hemiptera, Corixidae) in south-west Spain. Aquatic sciences. 2016; 78(4), 779–791.

[38] Aykut M, Zawal A, Esen Y, Erman O, et al First record of larvae of the water mite *Hydrachna processifera* Piersig, 1895 from Turkey (Acari, Hydrachnidia, Hydrachnidae). ZooKeys. 2018; no 738, p. 89. 10.3897/zookeys.738.21021 PMC5904403 29670424

[39] Davids C, Di Sabatino A, Gerecke R, Gledhill T, Smit H, van der Hammen H. Acari: hydrachnidia In: Gerecke R (ed) Freshwater Fauna of Central Europe. 2006; vol 7/2-1. Spektrum Akademischer Verlag, München, pp 241–388

[40] Lanciani CA. Three species of Eylais (Acari: Eylaidae) parasitic on aquatic Hemiptera. Transactions of the American Microscopical Society. 1969; 356–365

[41] Nielsen GJ, Davids C. Contributions to the knowledge of the morphology and biology of the larvae of four European Eylais species (Acari, Hydrachnellae). Acarologia. 1976.

[42] Lorenzo-Carballa MO, Beatty CD, Haitlinger R, Valdecasas AG, Utzeri C, Vieira V, et al Larval aquatic and terrestrial mites infesting parthenogenetic *Ischnura hastata* (Odonata: Coenagrionidae) from the Azores islands. Experimental and Applied Acarology. 2011; 54(3) 225–241 10.1007/s10493-011-9437-5 21380754

[43] Davids C. The water mite *Hydrachna conjecta* Koenike (Acari: Hydrachnellae), bionomics and relation to species of Corixidae (Hemiptera). Neth J Zool. 1973;23, pp. 363–429

[44] Smith BP. Host-parasite interaction and impact of larval water mites on insects. Annual review of entomology. 1988; 33(1) 487–507

[45] Marcogliese DJ, Pietrock M. Combined effects of parasites and contaminants on animal health: parasites do matter. Trends in parasitology 27.3 2011; 123–130. 10.1016/j.pt.2010.11.002 21144800

[46] Lanciani CA, May PG. Parasite-mediated reductions in the growth of nymphal backswimmers. Parasitology. 1982; 85(1) 1–7

[47] Randolt K, Gimple O, Geissendörfer J, Reinders J, Prusko C, Mueller MJ, et al Immune‐related proteins induced in the hemolymph after aseptic and septic injury differ in honey bee worker larvae and adults. Archives of Insect Biochemistry and Physiology: Published in Collaboration with the Entomological Society of America. 2008; 69(4):155–67. 10.1002/arch.20269 18979500

[48] Davids C, Schoots CJ. The influence of the water mite species Hydrachna conjecta and H. cruenta (Acari, Hydrachnellae) on the egg production of the Corixidae Sigara striata and Cymatia coleoptrata (Hemiptera). Internationale Vereinigung für theoretische und angewandte Limnologie: Verhandlungen. 1975; 12 1;19(4):3079–82.

[49] Lanciani CA. Parasite‐Induced Alterations in Host Reproduction and Survival. Ecology. 1975; 56(3) 689–695

[50] Crisp DT. Hydracarines and nematodes parasitizing *Corixa scotti* (D and S) (Hemiptera) in western Ireland. The Irish Naturalists' Journal. 1959; 88–92

[51] Stevens M, Greven H. Food and feeding behaviour of deutonymphs and adults of the water mite *Hydrachna skorikowi* (Acari: Hydrachnellae), with notes on the structure of their mouthparts In Ecology and Evolution of the Acari. 1999; (pp 381–387) Springer Dordrecht

[52] Céspedes V, Coccia C, Carbonell JA, Sánchez MI, Green AJ. The life cycle of the alien boatman *Trichocorixa verticalis* (Hemiptera, Corixidae) in saline and hypersaline wetlands of south-west Spain. Hydrobiologia. 2018; 1–16. 10.1007/s10750-018-3782–x

[53] Poulin R. Toxic pollution and parasitism in freshwater fish. Parasitology Today. 1992; 8(2) 58–61 15463572 10.1016/0169-4758(92)90090-o

[54] Overstreet RM. Parasitic diseases of fishes and their relationship with toxicants and other environmental factors. Pathobiology of marine and estuarine organisms. 1993; 111–156

[55] Blanar CA, Munkittrick KR, Houlahan J, MacLatchy DL, Marcogliese DJ. Pollution and parasitism in aquatic animals: a meta-analysis of effect size. Aquatic Toxicology. 2009; 4; 93(1):18–28. 10.1016/j.aquatox.2009.03.002 19349083

[56] Sures B, Nachev M, Selbach C, Marcogliese DJ. Parasite responses to pollution: what we know and where we go in ‘Environmental Parasitology’. Parasites & vectors. 2017; 10(1) 65 28166838 10.1186/s13071-017-2001-3 PMC5294906

[57] Studer A, Poulin R. Effects of salinity on an intertidal host–parasite system: Is the parasite more sensitive than its host?. Journal of Experimental Marine Biology and Ecology. 2012; 412 110–116

[58] Hall MD, Vettiger A, Ebert D. Interactions between environmental stressors: the influence of salinity on host–parasite interactions between *Daphnia magna* and *Pasteuria ramose*. Oecologia. 2013; 171(4) 789–796 10.1007/s00442-012-2452-3 23001624

[59] Tones PI, Hammer UT. Osmoregulation in *Trichocorixa verticalis interiores* Sailer (Hemiptera, Corixidae)—an inhabitant of Saskatchewan saline lakes, Canada. Canadian Journal of Zoology. 1975; 53(9), 1207–1212.

[60] Van de Meutter F, Trekels H, Green AJ. The impact of the North American waterbug *Trichocorixa verticalis* (Fieber) on aquatic macroinvertebrate communities in southern Europe. Fundamental and Applied Limnology/Archiv für Hydrobiologie. 2010; 177(4) 283–292

[61] Carbonell JA, Millán A, Green AJ, Céspedes V, Coccia C, Velasco J. What traits underpin the successful establishment and spread of the invasive water bug *Trichocorixa verticalis verticalis*?. Hydrobiologia. 2016; 768(1):273–86.

[62] Carbonell JA, Millán A, Velasco J. Concordance between realised and fundamental niches in three Iberian Sigara species (Hemiptera: Corixidae) along a gradient of salinity and anionic composition. Freshwater biology. 2012; 57(12), 2580–2590.

[63] Scudder GGE. Water-boatmen of saline waters (Hemiptera: Corixidae) In Marine Insects, editor Cheng L., North-Holland Publishing Co 1976; N.Y pp. 263–289

[64] Bradley TJ. Saline-water insects: ecology, physiology and evolution In: Aquatic Insects: Challenges to Populations (Eds. Lancaster J. & Briers R.A.). 2008; pp. 20–35. CAB International 2008, Oxfordshire OX10 8DE, UK

[65] Heine-Fuster I, Vega-Retter C, Sabat P, Ramos-Jiliberto R. Osmoregulatory and demographic responses to salinity of the exotic cladoceran *Daphnia exilis*. Journal of Plankton Research. 2010; 32(10) 1405–1411

[66] Oren A. Bioenergetic aspects of halophilism. Microbiology and molecular biology reviews. 1999; 63(2) 334–348 10357854 10.1128/mmbr.63.2.334-348.1999 PMC98969

[67] Åbro A. The effects of parasitic water mite larvae (Arrenurus spp.) on zygopteran imagoes (Odonata). Journal of Invertebrate Pathology. 1982 5 1;39(3):373–81.

[68] Fairn ER, Schulte-Hostedde AI, Alarie Y. Water mite parasitism is associated with body condition and sex of the whirligig beetle *Dineutus nigrior* (Coleoptera: Gyrinidae) Ecoscience. 2008; 15 3; 327–331

