# Supplementary material for: Water boatman survival and fecundity are related to ectoparasitism and salinity stress
Source: PLoS One. 2019 Jan 16;14(1):e0209828. doi: 10.1371/journal.pone.0209828 (PMC6334896; doi:10.1371/journal.pone.0209828)
Supplement: S1 Table — Physico-chemical characteristics of Laguna Dulce from RBD (Doñana Biological Reserve) on 09/06/2014. (DOCX) [file pone.0209828.s001.docx]

**S1 Table:** Physico-chemical characteristics of Laguna Dulce from RBD (Doñana Biological Reserve) on 09/06/2014.

| pH | TºC | Cond. (µS.cm ^-1^) | Sal. (g.l^-1^) | Turb.  FTU | Mean depth  (cm) | NH_4_^+^ (µg N /l) | PO _4_ ^3-^ (µg P/l) | NO_2_^-^ (µg N /l) | NO_3_^-^ (µg N /l) | TP (µg/l) | TN (µg/l) | Chla (µg/L) |
| --- | --- | --- | --- | --- | --- | --- | --- | --- | --- | --- | --- | --- |
| 6.30 | 24.10 | 1915 | 0.80 | 8.44 | 33.00 | 27.98 | 32.34 | 1.265 | 0.896 | 175.6 | 4421 | 15.25 |
