# Supplementary material for: Water boatman survival and fecundity are related to ectoparasitism and salinity stress
Source: PLoS One. 2019 Jan 16;14(1):e0209828. doi: 10.1371/journal.pone.0209828 (PMC6334896; doi:10.1371/journal.pone.0209828)
Supplement: S2 Table — List of species of Hemiptera and the abundance of each species sampled from Dulce pond (RBD) on 09/06/2014. (DOCX) [file pone.0209828.s002.docx]

**S2Table:** List of species of Hemiptera and the abundance of each species sampled from Laguna Dulce (RBD) on 09/06/2014.

Corixidae

*Corixa panzeri* (N=6)

*Corixa affinis* (N=33)

*Corixa* sp. (N=2)

*Micronecta scholtzi* (N=1)

*Sigara lateralis* (N=2063)

*Sigara stagnalis* (N=42)

*Sigara scripta* (N=11)

*Trichocorixa verticalis* (N=1)

Gerridae

*Gerris thoracicus* (N=22)

Naucoridae

*Naucoris maculatus* (N=1)

Notonectidae

*Anisops sardeus* (N=16)

*Notonecta glauca* (N=36)

Pleidae

*Plea minutissima* (N=5)
