# Supplementary material for: Water boatman survival and fecundity are related to ectoparasitism and salinity stress
Source: PLoS One. 2019 Jan 16;14(1):e0209828. doi: 10.1371/journal.pone.0209828 (PMC6334896; doi:10.1371/journal.pone.0209828)
Supplement: S3 Table — Prevalence of water mites in adults and larvae of corixids that were sampled in the field on the same date and in the same locality as samples collected for experimental analyses (Dulce pond on 09/06/2014). H (Hydrachna skorikowi), E (Eylais infundibulifera)). (DOCX) [file pone.0209828.s003.docx]

**S3 Table:** Prevalence of water mites in adults and nymphs of corixids that were sampled in the field on the same date and in the same locality as samples collected for experimental analyses (Dulce pond on 09/06/2014). H (*Hydrachna skorikowi*), E (*Eylais infundibulifera*)).

|  |  |  |  | Prev% | | | N | |
| --- | --- | --- | --- | --- | --- | --- | --- | --- |
| **Genera** | **Species** | **Sex** | **Stage** | **H** | **E** | **TOT** | **Parasitized** | **Unparasitized** |
| *Sigara* | *lateralis* | female | adult | 1.264 | 0.564 | 1.829 | 16 (11H+5E) | 859 |
| *Sigara* | *lateralis* | male | adult | 0.760 | 0.339 | 1.094 | 13 (9H+4E) | 1175 |
| *Sigara* | *lateralis* |  | II | 1.299 |  | 1.299 | 1H | 76 |
| *Sigara* | *lateralis* |  | III | 1.754 |  | 1.754 | 2H | 114 |
| *Sigara* | *lateralis* |  | IV |  |  |  | _ | 60 |
| *Sigara* | *lateralis* |  | V |  |  |  | _ | 116 |
| *Corixa* | *affinis* | female | adult |  |  |  | _ | 16 |
| *Corixa* | *affinis* | male | adult |  |  |  | _ | 17 |
