# Supplementary material for: Water boatman survival and fecundity are related to ectoparasitism and salinity stress
Source: PLoS One. 2019 Jan 16;14(1):e0209828. doi: 10.1371/journal.pone.0209828 (PMC6334896; doi:10.1371/journal.pone.0209828)
Supplement: S4 Table — Effects of salinity treatments, infection status (Hydrachna skorikowi and uninfected), sex (female and male) and their interactions on survival times in Corixa affinis adults. Salinity treatments were 0.5, 5, 10 and 15 g/l and water from the collection site as a control (0.8 g/l). The table shows for each term in the design matrix the estimated coefficient βj^ (coef), the relative risk exp (βj^) (exp (coef)), the standard error, the z-value and the corresponding P-value. Each P-value provides a test for the difference of each level with respect to the baseline. The overall P-value for factors with more than two levels (i.e. salinity) and for the interaction infection status*salinity, is obtained through the Wald test and is shown under the table. (DOCX) [file pone.0209828.s004.docx]

**S4 Table: Survival analysis (Cox proportional hazard regression) for *Corixa affinis* adults.** Effects of salinity treatments, infection status (*Hydrachna skorikowi* and uninfected), sex (female and male) and their interactions on survival times in *Corixa affinis* adults. Salinity treatments were 0.5, 5, 10 and 15 g/l and water from the collection site as a control (Ctrl: 0.8 g/l). The table shows for each term in the design matrix the estimated coefficient *β̂_j_* (coef), the relative risk exp(*β̂_j_*) (exp (coef)), the standard error, the *z*-value and the corresponding *P*-value. Each *P*-value provides a test for the difference of each level with respect to the baseline. The overall *P*-value for factors with more than two levels (i.e. salinity) and for the interaction infection status*salinity, is obtained through the Wald test and is shown under the table.

| **Effect** | | **Level of effect** | **coef** | **exp (coef)** | **s.e. (coef)** | **Z-value** | **P-value (>\|z\|)** |
| --- | --- | --- | --- | --- | --- | --- | --- |
| Infection status | | Unparasitized | -1.607 | 0.200 | 0.50411 | -3.189 | **P<0.001** |
| Sex | | Male | -0.165 | 0.847 | 0.249 | -0.661 | ns |
| Salinity (g/l) | | Ctrl | -0.242 | 0.785 | 0.398 | -0.607 | ns |
|  | | 5 | -0.828 | 0.437 | 0.455 | -1.820 | 0.062 |
|  | | 10 | 1.410 | 4.097 | 0.478 | 2.948 | **0.003** |
|  | | 15 | 1.504 | 4.498 | 0.472 | 3.188 | **0.0014** |
|  | | 20 | 3.774 | 43.55 | 0.551 | 6.840 | **P<0.001** |
| Infection status*Sex | | Unparasitized* Male | 0.755 | 2.123 | 0.360 | 2.096 | **0.036** |
| Infection status*Salinity | | Unparasitized *Ctrl | 0.445 | 1.562 | 0.555 | 0.803 | ns |
|  | | Unparasitized *5 | 1.823 | 6.189 | 0.655 | 2.783 | **0.005** |
|  | | Unparasitized *10 | 0.610 | 1.849 | 0.649 | 0.946 | ns |
|  | | Unparasitized *15 | 0.181 | 1.198 | 0.648 | 0.279 | ns |
|  | | Unparasitized *20 | 0.064 | 1.065 | 0.683 | 0.092 | ns |
|  | Concordance= 0.793 (s.e. = 0.031). Rsquare= 0.582 (max possible= 1 ) | | | | | | |

Overall P-value for Salinity variable and Infection status*Salinity. Wald test “Salinity”; X^2^ = 81.3, df = 5, P(> X^2^) < **0.00001**; “Infection status*Salinity”; X^2^ = 10.7, df = 5, P(> X^2^) = 0.058.
