# Supplementary figures and images for: Water boatman survival and fecundity are related to ectoparasitism and salinity stress
Source: PLoS One. 2019 Jan 16;14(1):e0209828. doi: 10.1371/journal.pone.0209828 (PMC6334896; doi:10.1371/journal.pone.0209828)

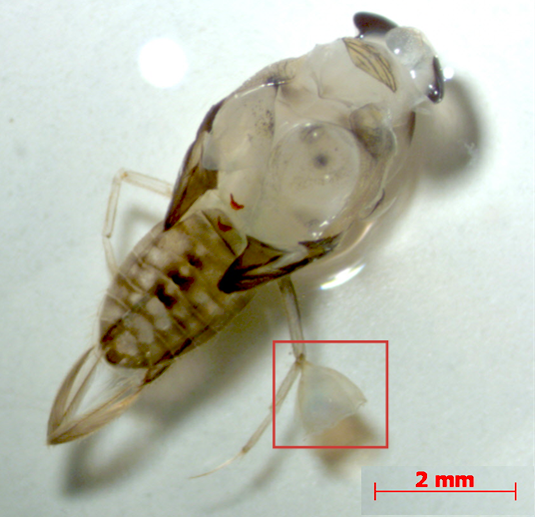

Supplement: S1 Fig — A failed moult of Sigara lateralis apparently due to the consequences of mite parasitism. The individual died in the act of moulting from larva stage V to the adult stage. The exuvia from a water mite Hydrachna skorikowi is highlighted in an orange box (this was reddish. but is discoloured after preservation in alcohol). The mite moulted successfully into a free-living adult. Credit: Vanessa Céspedes. (TIF) [file pone.0209828.s005.tif]

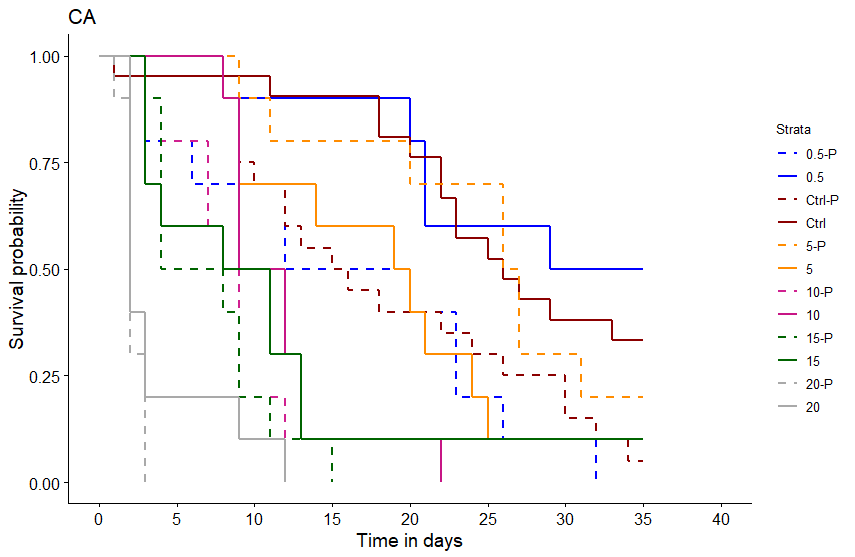

Supplement: S2 Fig — Survival time for adult female and male Corixa affinis (CA) with and without mite parasites Hydrachna skorikowi under different salinity treatments. C.W. represents water from the collection site (0.8 g/l). (TIFF) [file pone.0209828.s006.tiff]
